# Neuropilin-1 as Therapeutic Target for Malignant Melanoma

**DOI:** 10.3389/fonc.2015.00125

**Published:** 2015-06-03

**Authors:** Grazia Graziani, Pedro M. Lacal

**Affiliations:** ^1^Department of Systems Medicine, University of Rome “Tor Vergata”, Rome, Italy; ^2^Laboratory of Molecular Oncology, “Istituto Dermopatico dell’Immacolata”, Istituto di Ricovero e Cura a Carattere Scientifico, Rome, Italy

**Keywords:** neuropilin-1, melanoma, peptidomimetics, cell-penetrating peptides, T regulatory cells, angiogenesis, metastasis

## Abstract

Neuropilin-1 (NRP-1) is a transmembrane glycoprotein that acts as a co-receptor for various members of the vascular endothelial growth factor (VEGF) family. Its ability to bind or modulate the activity of a number of other extracellular ligands, such as class 3 semaphorins, TGF-β, HGF, FGF, and PDGF, has suggested the involvement of NRP-1 in a variety of physiological and pathological processes. Actually, this co-receptor has been implicated in axon guidance, angiogenesis, and immune responses. NRP-1 is also expressed in a variety of cancers (prostate, lung, pancreatic, or colon carcinoma, melanoma, astrocytoma, glioblastoma, and neuroblastoma), suggesting a critical role in tumor progression. Moreover, a growing amount of evidence indicates that NRP-1 might display important functions independently of other VEGF receptors. In particular, in the absence of VEGFR-1/2, NRP-1 promotes melanoma invasiveness, through the activation of selected integrins, by stimulating VEGF-A and metalloproteinases secretion and modulating specific signal transduction pathways. This review is focused on the role of NRP-1 in melanoma aggressiveness and on the evidence supporting its use as target of therapies for metastatic melanoma.

## Introduction

**Figure 1:**
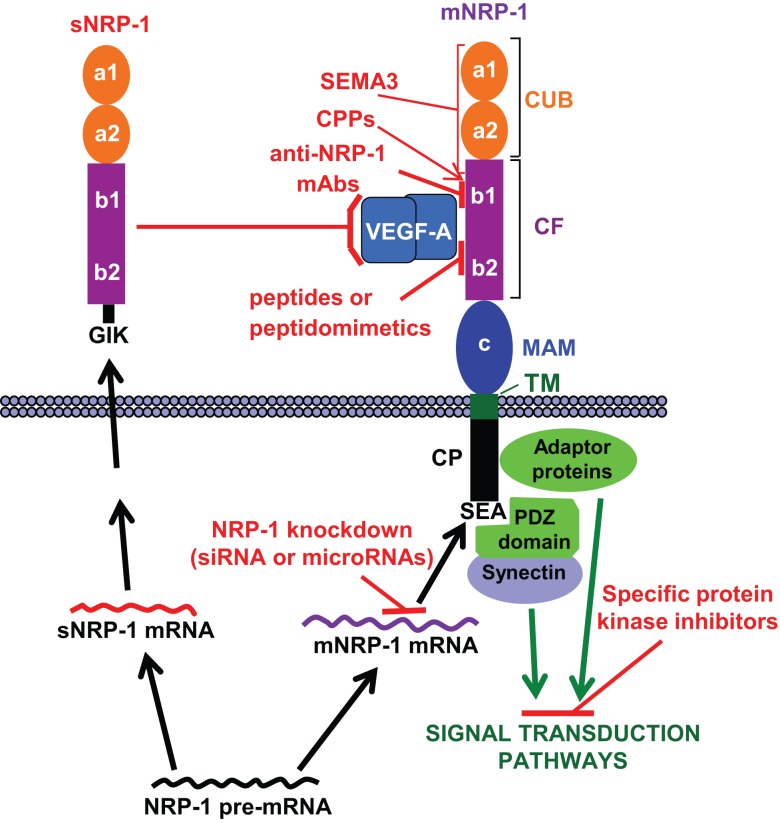
**NRP-1 structure and strategies to target its functions**. The extracellular region of membrane NRP-1 (mNRP-1) is divided into three domains: a1/a2, which is homologous to the complement proteins C1r/C1s, Uegf and Bmp-1 (CUB); b1/b2, which is homologous to the coagulation factors V and VIII (CF); c, which is homologous to meprin, A5 and receptor tyrosine phosphatase μ (MAM). The cytoplasmic tail (CP) lacks a catalytic activity, but contains a C-terminal SEA sequence that represents a consensus binding motif for proteins containing the PDZ (PSD-95, Dlg, ZO-1) domain (e.g., synectin), which promotes the formation of complexes with signaling components (15). The interaction of NRP-1 cytoplasmic tail with adaptor proteins may trigger different signal transduction pathways (see text). Soluble NRP-1 (sNRP-1) contains the extracellular a1/a2 and b1/b2 domains but lacks the c, transmembrane and cytoplasmic domains, ending with an intron-derived three amino acids sequence (GIK). NRP-1 ligands and interacting receptors include (16, 17): (a) class 3 semaphorins (SEMA3) that bind to the CUB and, partly, to the CF domains; (b) growth factors (VEGF-A/B/D/E, PlGF-2, HGF, TGFβ1, bFGF, PDGF), all binding to the CF domain; (c) membrane receptors (plexins, VEGFRs, PDGFR, TGFR) that interact with NRP-1 through its dimerization MAM and CF domains. HGFR interacts with NRP-1 CUB domain (18). The possible strategies to target NRP-1 function are indicated in red: (a) blockade of growth factor binding to membrane NRP-1 by sNRP-1; (b) induction of inhibitory signals by class 3 semaphorins binding to NRP-1; (c) blockade of VEGF-A binding to NRP-1 with monoclonal antibodies (mAbs), peptides or peptidomimetics; (d) knockdown of NRP-1 expression with small interfering RNAs (siRNA) or microRNAs; (e) delivery of therapeutic agents to NRP-1 expressing cells using cell-penetrating peptides (CPPs), which interact with the CF domain; (f) inhibition of the signal transduction pathways triggered by NRP-1 activation. Please, refer to the text for further details.

Neuropilin-1 (NRP-1) is a transmembrane glycoprotein, composed of a large N-terminal extracellular region, a short transmembrane domain and a small cytoplasmic tail (44 aa) (1) (Figure 1). It was originally identified as co-receptor for class 3 semaphorins, a family of molecules that provide repulsive or attractive signals for neurons (2, 3). Actually, NRP-1 was shown to be involved in neural crest migration and axon growth during the development of the nervous system by forming a complex with type-A plexin, a signal-transducing transmembrane receptor for class 3 semaphorins (4, 5). Studies on over-expression and/or ectopic expression of NRP-1 in chimeric mouse embryos or inactivation of the gene in mutant mice indicated that NRP-1 is required during embryogenesis not only for the neuronal guidance but also for the normal development of the cardiovascular system (6, 7). In fact, NRP-1 is expressed in endothelial cells, where it interacts with several members of the vascular endothelial growth factor (VEGF) family of angiogenic factors and some of their tyrosine kinase receptors enhancing the signaling and promoting angiogenesis (8–14).

Besides its critical role during embryogenesis, NRP-1 has important functions in the adult tissues, being involved in axon guidance (mediated by class 3 semaphorins), vascular endothelial sprouting (triggered by VEGFs), and immunosuppression [mediated by plasmocytoid dendritic cells (pDCs) and T regulatory cells (Tregs)]. Other ligands of NRP-1 include (Figure 1): transforming growth factor-β1 (TGF-β1) and its receptors, hepatocyte growth factor (HGF) and its receptor c-met, platelet-derived growth factor (PDGF) and its receptors, fibroblast growth factor (FGF), anti-thrombin III, and galectin-1 (16, 19). In addition, NRP-1 interacts with other transmembrane proteins such as αvβ3 and β1 integrin (20–23).

NRP-1 is also able to respond to some of its ligands even in the absence of the corresponding tyrosine kinase receptors. For instance, PDGF-B, through the interaction with NRP-1, controls the differentiation and recruitment of mesenchymal stem cells and stimulates the migration of smooth muscle cells (24–26). Moreover, placenta growth factor (PlGF, a member of the VEGF family) has been shown to promote the growth and survival of medulloblastoma after binding to NRP-1 (27). The ability of NRP-1 to initiate signal transduction pathways has been attributed to the interaction of its cytoplasmic tail with adaptor polypeptides, which activate downstream molecules, such as Akt or p130Cas/FAK, involved in cell proliferation, migration, survival, and invasion (26, 28, 29). Moreover, the interaction of NRP-1 with ABL1 promotes paxillin phosphorylation and actin remodeling, favoring cell motility *in vitro* and angiogenesis *in vivo* (30) (Figure 1).

In addition to the membrane form, a naturally occurring soluble NRP-1 protein (sNRP-1), containing only part of the extracellular domain, is generated by alternative splicing of the NRP-1 gene (Figure 1) (31, 32) and is thought to function as a natural inhibitor of the membrane NRP-1 by sequestering its ligands.

## NRP-1 in Tumor Progression: Role in Melanoma

NRP-1 is expressed not only in tumor-associated vessels but also in a variety of cancers suggesting a role in tumor progression. In a recent study utilizing carcinomas, NRP-1 has been detected in blood vessels in more than 98% of cases, whereas its expression in cancer varies depending on the tissue origin, histological sub-type and stage (33). Increased levels of NRP-1 correlate with tumor aggressiveness, advanced disease stage, and poor prognosis (19, 34). NRP-1 up-regulation appears to be associated with the tumor invasive behavior and metastatic potential (35), for instance in melanoma and breast cancer (9, 36). This receptor has been implicated in mediating the effects of VEGF-A and semaphorins on the proliferation, survival, and migration of cancer cells (36–42). NRP-1 is also expressed by various stromal cells, including fibroblasts, endothelial and immune cells, which can be activated by growth factors different from VEGF-A and contribute to tumor progression. In fact, although the cancer promoting effects of NRP-1 have often been attributed to an enhancement of VEGF receptors (VEGFR)-2 activation in response to VEGF-A, some tumors express NRP-1 but neither VEGFR-1 nor VEGFR-2 (26, 43, 44).

A large number of human melanoma cell lines, derived from primary and metastatic lesions, secrete VEGF-A and express its receptors, including NRP-1 (45). NRP-1 enhances the activation of a VEGF-A/VEGFR-2 autocrine loop, which promotes the invasion of melanoma cells into the extracellular matrix (46), through the up-regulation of VEGF-A and metalloproteinases secretion (29, 47). Moreover, NRP-1 over-expression provides human melanoma cells with an increased *in vivo* growth rate (48).

NRP-1 might be also involved in the effects of PlGF on melanoma cells. This angiogenic factor, has been detected in specimens from melanoma patients by immunohistochemical staining, is secreted by melanoma cells and promotes *in vitro* extracellular matrix invasion and matrix metalloproteinases secretion (45, 49). In a transgenic murine model, the over-expression of PlGF in the skin significantly favored the growth and metastasis to the lungs of syngeneic melanoma cells orthotopically implanted in the skin (49). Moreover, PlGF plays a role in the resistance of melanoma to temozolomide, an anticancer agent used for the treatment of the metastatic disease, through a mechanism involving NF-kB (50). Interestingly, melanoma cells expressing NRP-1 but lacking other VEGF-A or PlGF receptors, specifically responded to PlGF in a chemotactic assay (51), suggesting that PlGF may perform at least some of its functions through activation of NRP-1 dependent pathways.

**Figure 2:**
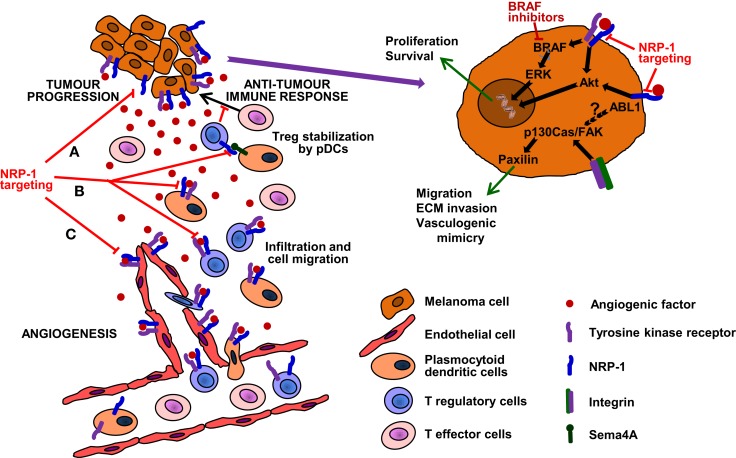
**Targeting of NRP-1 in the treatment of melanoma**. The targeting of this receptor is expected to result in therapeutic benefit by at least three mechanisms (see text for details): **(A)** *Decrease of melanoma aggressiveness, metastatic potential and chemoresistance*. NRP-1 targeting, besides reducing the invasiveness and vasculogenic mimicry that confer melanoma cells an aggressive and metastatic phenotype, might also counteract resistance to inhibitors of mutated BRAF. **(B)** *Enhancement of antitumor immune responses*. NRP-1 targeting may inhibit the recruitment of pDCs and Tregs, reduce immune suppression against melanoma and synergize with immunotherapies, delaying tumor progression and development of resistance. **(C)** *Inhibition of angiogenesis*. Melanoma growth and dissemination is dependent on angiogenesis and NRP-1 cooperates in the signal transduction of tyrosine kinase receptors activated by angiogenic factors and involved in the formation of new vessels (53). Moreover, NRP-1 over-expression is involved in tumor resistance to anti-angiogenic therapies targeting VEGF-A and VEGFR-1/2 (54). Therefore, the targeting of NRP-1 may inhibit neovessel formation and counteract resistance to anti-VEGF-A therapies.

Highly malignant cells, because of their ability to de-differentiate and acquire characteristics of other cell types, may form *de novo* vascular networks (vasculogenic mimicry), contributing to new vessel formation. Vasculogenic mimicry favors tumor growth and invasion and predicts poor prognosis in melanoma patients (52). It has been recently demonstrated that NRP-1 expression in melanoma cells increases their aggressiveness and ability to form tubule-like structures (47). These NRP-1-mediated effects require the activation of specific integrins. In particular, αvβ5 integrin favors cell adhesion to vitronectin and collaborates with NRP-1 in the development of an invasive and vasculogenic mimicry phenotype (47). In this context, NRP-1 has been shown to complex with the intracellular kinase ABL1 after adhesion of endothelial cells to fibronectin, resulting in phosphorylation of the focal adhesion component paxillin and promotion of cell migration (30). If confirmed in NRP-1 expressing melanoma cells, this pathway might also contribute to tumor aggressiveness (Figure 2).

NRP-1 has been indicated as a promoter of epithelial-mesenchymal transition, a critical step in tumor invasion and disease progression. A similar process of phenotype switching has been reported in melanoma and implicated in promotion to a metastatic state, providing further evidence of NRP-1 involvement in multiple oncogenic functions (55, 56).

This evidence supports the hypothesis that NRP-1 might represent a suitable target for anti-melanoma therapies. However, since this protein interacts with a number of tumor-associated molecules, further studies are required to define its precise mechanisms of action in melanoma progression.

## NRP-1 as Therapeutic Target

Several strategies have been explored to counteract the tumor promoting effects of NRP-1 function, employing different tools (Figure 1): recombinant sNRP-1, class 3 semaphorins, monoclonal antibodies (mAbs), peptides and peptidomimetics, small interfering RNAs (siRNAs) or microRNAs.

### Administration of sNRP-1

sNRP-1, functioning as natural ligand trap, inhibits the interaction of VEGF-A or other growth factors with their specific receptors and with membrane NRP-1 expressed by tumor or normal cells (32, 57, 58). Indeed, the injection of an adenovirus encoding for sNRP-1 significantly inhibited neoangiogenesis and prolonged the survival of leukemia-bearing mice (59). sNRP-1 also decreased the invasiveness of human non-small cell lung cancer cells *in vitro* (41) and its over-expression inhibited breast cancer cell migration (32, 60).

Interestingly, following administration of an anti-NRP-1 mAb (see below) that specifically recognizes the coagulation factors domain of this receptor, an increase in circulating NRP-1 in the serum of treated patients was observed (61). In this case, circulating NRP-1 is likely the result of membrane NRP-1 shedding and may contribute to enhance the efficacy of the anti-NRP-1 mAb by sequestering VEGF-A.

Another mechanism by which sNRP-1 might modulate VEGF-A signal transduction is the formation of a complex with VEGF-A bound to VEGFR-2. A similar mechanism has been recently described in murine B16 melanoma cells, where tumor-associated NRP-1 can prevent VEGFR2/VEGF-A internalization and signaling in endothelial cells through a *trans* complex formation, suppressing tumor initiation and angiogenesis (62).

### Over-expression of class 3 semaphorins

Semaphorins and VEGF-A seem to compete for NRP-1 binding, although they interact with different domains of the receptor. Indeed, semaphorins are responsible for inhibition of cancer cell proliferation and induction of apoptosis, whereas VEGF-A enhances angiogenesis and tumor growth. Since semaphorins such as Sema3B and Sema3F are often down-regulated in tumor cells (63), over-expression of Sema3 genes may represent a promising therapeutic strategy to antagonize VEGF-A-mediated effects (64). However, exogenous administration of Sema3A induced proteinuria by disrupting podocyte foot processes in the kidney (65).

### Blockade of VEGF-A binding with anti-NRP-1 monoclonal antibodies

A high-affinity mAb targeting the coagulation factors domain of NRP-1 (anti-NRP-1B) has been shown to inhibit VEGF-A-induced migration of human endothelial cells and tumor formation in animal models avoiding VEGF-A-binding to NRP-1 (66–69). This antibody also potentiates the effects of an anti-VEGF-A therapy (68). These findings have led to speculate that the combination of anti-NRP-1 and anti-VEGF agents might improve the survival of patients with advanced malignancies. Moreover, the anti-NRP-1B antibody was found to directly inhibit breast cancer cell proliferation, adhesion to fibronectin and formation of NRP-1/α5β1 integrin complexes, as well as phosphorylation of FAK and p130cas (70). Finally, it enhanced chemosensitivity of human non-small-cell lung, kidney, prostate cancer, and other carcinoma cells, by interfering with integrin-dependent survival pathways (43).

The majority of anti-angiogenic therapies developed so far target the VEGF-A signaling by blocking VEGF-A and inhibiting VEGFR-1 and/or VEGFR-2 activation (71). However, primary and acquired resistance to the available anti-angiogenic therapies is commonly reported. Thus, other anti-angiogenic approaches that affect additional signal transduction pathways are under investigation. In this context, a human mAb (i.e., MNRP1685A) that blocks the binding of VEGF-A, VEGF-B, and PlGF to NRP-1 is currently under evaluation in clinical trials. This antibody does not affect the binding of Sema3A to the CUB domain that regulates neurogenesis. Phase I studies with MNRP1685A in patients with advanced solid tumors showed that this antibody is generally well-tolerated as single agent, but it has a modest clinical activity (72). However, when co-administered with bevacizumab and paclitaxel, it caused a high rate of clinically significant proteinuria, not supporting further testing of MNRP1685A associated with the anti-VEGF-A antibody (73).

### Blockade of VEGF-A binding to NRP-1 with specific peptides and peptidomimetics

Differently from semaphorins, VEGF-A binds exclusively to the NRP-1 coagulation factors domain (74). This allows the selective targeting of VEGF-A/NRP-1 interaction without affecting the binding of class 3 semaphorins. Indeed, several specific peptides and peptidomimetics, capable of competitively inhibiting the VEGF-A/NRP-1 interaction, exerted anti-angiogenic activity through down-regulation of VEGF-A signaling, as demonstrated by the reduced VEGFR-2 tyrosine phosphorylation and *in vitro* tubule-like formation (75–77). These molecules induced apoptosis in NRP-1-expressing breast cancer cells (39) and decreased *in vivo* tumor growth (77).

Peptides are not considered viable drugs, but they provide an appropriate starting point for the structure-based design of peptidomimetics and small molecule inhibitors. An example is the potent peptidomimetic compound _D_LPR, a tripeptide resistant to proteolysis generated by the amino acid retroinversion method (substitution of d- for l-amino acids and sequence reversal) (78). This peptide interacted with NRP-1 and exhibited anti-angiogenic activity in different *in vivo* animal models of cancer.

The molecular design of a small molecule ligand that fits into the VEGF-A binding site of the NRP-1 coagulation factors domain has been reported (79). This inhibitor, denoted as EG00229, derives from the previously characterized bicyclic peptide EG3287 that corresponds to the C-terminal 28-residue segment of VEGF-A (75). EG00229 inhibited VEGF-A binding to NRP-1, decreased VEGFR-2 phosphorylation and the migration of lung carcinoma cells *in vitro*. Moreover, it enhanced tumor sensitivity to the cytotoxic effects of paclitaxel and 5-fluorouracil.

Other NRP-1 antagonists designed on the basis of a previously described NRP-1 inhibitory peptide have been recently produced (80), but data on their efficacy in preclinical *in vivo* tumor models are not available yet.

### NRP-1 knockdown with small interfering RNAs or microRNAs

Small interfering RNAs have also been utilized to target NRP-1, resulting in a significant reduction of the growth, angiogenesis and metastasis formation in various human tumor models, such as hepatocellular carcinoma (81, 82), acute myeloid leukemia (83), and lung cancer (41). NRP-1 silencing with specific siRNA also impaired the activity of several growth factors (84) and increased the sensitivity to chemotherapeutic agents (e.g., 5-fluorouracil, paclitaxel, and cisplatin) (43).

NRP-1 has been shown to be the target of several microRNAs (miR), such as miR-9, miR-181b, and miR-320, which modulate angiogenesis and tumor invasion (85, 86). Hence, it has been suggested that these microRNAs might be good candidates for cancer treatment. In particular, the anti-angiogenic microRNA miR-320a, by targeting NRP-1, suppressed the *in vitro* migration, adhesion and tubule formation by vascular endothelial cells, and reduced *in vivo* tumor angiogenesis and colon cancer cell migration and invasion (86, 87). These findings support the possible development of microRNA-based agents as anti-angiogenic and/or anticancer drugs.

### Cell-penetrating peptides

In the search for cell-penetrating peptides (CPPs), the screening of phage peptide libraries led to the observation that many CPPs have a C-terminal R/KXXR/K consensus sequence, referred to as the C-end rule (CendR) motif (88, 89). Peptides with these characteristics appear to bind to the electronegative pocket of the coagulation factors domain of NRP-1, which mediates their rapid internalization into NRP-1-expressing cells. Since NRP-1 is frequently expressed in cancer cells, this NRP-1 activity is being explored for the targeted delivery of therapeutic and diagnostic agents (90, 91). Thus, CPPs appear particularly valuable to allow cell internalization of high molecular weight drugs that cannot cross the plasma membrane and to selectively target tumor tissues minimizing systemic toxicity.

Tumor-homing cyclic peptides, designated iRGD, are characterized by their ability to attach to RGD-binding integrins (88, 92). These compounds are cleaved on the membrane of tumor cells by a furin-like protease, which exposes a CendR motif (RGDK/R), allowing their interaction with NRP-1 and the internalization of the complex, along with a potential peptide-linked cargo. Conjugation of these peptides with imaging or chemotherapeutic agents enhanced tumor detection and the activity of anticancer therapies (88, 89, 92). For instance, iRGD-modified and doxorubicin-loaded sterically stabilized liposomes exhibited high distribution in B16 melanoma cells, and exerted antitumor and anti-angiogenic effects, with low systemic toxicity (93). Furthermore, the iRGD peptides induced vascular leakage, allowing extensive tumor penetration of the peptide, attached cargo and co-injected drug (92).

Moreover, nanoparticles carrying a therapeutic p53-stabilizing peptide alongside with the NRP-1-targeting peptide, showed promising *in vitro* anticancer activity (94), suggesting the potential applicability of this technology in different fields such as imaging, diagnosis, and combination therapies.

## Concluding Remarks

Cutaneous melanoma is an extremely aggressive cancer with high metastatic potential. Actually, melanoma’s ability to metastasize to distant organs is the primary cause of human skin cancer-related deaths. The identification of molecular mechanisms associated with the acquisition of a metastatic phenotype by melanoma cells is, therefore, of great importance for the design of more efficient therapies. In this context, three factors are crucial for melanoma progression: (1) formation of new blood vessels from the pre-existing vasculature (angiogenesis); (2) increased ability of tumor cells to invade the extracellular matrix and to form capillary-like structures (vasculogenic mimicry); (3) tumor evasion from the control of the immune system. NRP-1 is involved in all these biological processes, being expressed in endothelial, highly aggressive melanoma and immune cells. Thus, the targeting of NRP-1 seems to be a valuable strategy for combination therapies with, BRAF inhibitors, immunomodulating, or anti-angiogenic agents (Figure 2).

BRAF inhibitors target specific mutations of BRAF in the kinase domain, which are present in about 50% of melanomas and cause over-activation of the mitogen-activated protein kinase (MAPK)/extracellular-signal-regulated kinase (ERK) pathway, involved in cell proliferation/survival. However, responses to BRAF inhibitors are short-lived, due to the development of different mechanisms of resistance that lead to the recovery of the MAPK signaling or the activation of alternative pathways, such as PI3K/AKT/mTOR [reviewed in Ref. (95)]. In melanoma cells, NRP-1 has been shown to activate signal transduction pathways involving AKT (29). Moreover, NRP-1-dependent pathways described in endothelial cells or other tumor models (26, 28, 30, 70) might be active in melanoma and contribute to BRAF inhibitor resistance. Thus, inhibition of NRP-1 may prevent the activation of compensatory mechanisms that stimulate melanoma cell proliferation and limit the efficacy of BRAF inhibitors (Figure 2A).

Immunotherapy with immune checkpoint inhibitors represents an important advancement in the treatment of metastatic melanoma (96). These agents increase immune responses by enhancing effector T cell functions. However, melanoma may evade the control of immune system by several mechanisms, including the activation of tumor-infiltrating Tregs (97). NRP-1 is expressed in pDCs and in a subset of Tregs and favors the transendothelial migration of these cells in response to angiogenic factors produced by the tumor (98, 99). Tregs recruited in the tumor tissue suppress immune responses by inhibiting the proliferation of effector T cells specific for tumor-associated antigens. In fact, NRP-1 deficiency in Tregs impairs melanoma growth (100). The pDCs are one of the two main types of dendritic cells and are regarded as an unfavorable prognostic factor in melanoma, since they accumulate within the melanoma microenvironment and play a predominantly immunosuppressive role (101). Indeed, pDCs promote the differentiation and modulate the function of Tregs by mechanisms involving also the interaction of the NRP-1 present in Tregs with the transmembrane semaphorin 4A (Sema4A) expressed by pDCs (102, 103). However, the precise functional significance of Sema4A in physiological and pathological immune responses remains to be determined. To this regard, it has been suggested that Sema4A/NRP-1 interaction increases the stability and survival of intra-tumoral Tregs, whereas it is dispensable for the maintenance of immune homeostasis (103).

Thus, targeting NRP-1 would result in decrease of the immune suppressive activity of pDCs and Tregs against melanoma, likely without inducing autoimmunity. Moreover, inhibition of NRP-1 may synergize with immune checkpoint inhibitors, delaying tumor progression and development of resistance (Figure 2B).

Finally, the targeting of NRP-1 may not only inhibit neovessel formation, but also counteract resistance to anti-VEGF-A therapies. In fact, NRP-1 over-expression is one of the pro-angiogenic signaling pathways involved in the development of resistance to the current anti-angiogenic therapies targeting VEGF-A and VEGFR-1/2 (54) (Figure 2C).

Inhibition of NRP-1 function may also result in modulation of signal transduction pathways triggered by growth factors other than VEGF-A, such as PDGF, FGF, EGF, and HGF, which are implicated in tumor progression and are capable of binding NRP-1. Indeed, the results observed using therapies that prevent the binding of VEGF-A to NRP-1 can be likely attributed also to the blockade of the interaction of NRP-1 with ligands that share with VEGF-A the same NRP-1 binding site.

Although over-expressed in melanoma, NRP-1 has a widespread expression in normal adult tissues. This might explain the quick drop of the MNRP1685 antibody concentration in the serum, observed in preclinical and phase I clinical studies, due to a significant target-mediated clearance (72, 104). Thus, adverse effects can be expected from inhibition of NRP-1 physiological functions. Indeed, NRP-1 is required for axon guidance, angiogenesis, immunity and regulation of the actin cytoskeleton in podocytes of the Bowman’s capsule in the kidney (105). Interestingly, no NRP-1 related toxicity, such as neurotoxicity or nephrotoxicity, was observed when MNRP1685 was used as single agent, whereas increased proteinuria, as a potential damage of podocyte function, was reported in combination with bevacizumab (73).

A better understanding of NRP-1 contribution to intracellular signal transduction mechanisms and the design of molecules that impair the binding of specific ligands, involved in tumor progression, will provide additional opportunities for the development of new therapeutic approaches to target NRP-1 in melanoma and to limit systemic toxicity.

## Conflict of Interest Statement

The authors declare that the research was conducted in the absence of any commercial or financial relationships that could be construed as a potential conflict of interest.

## Abbreviations

NRP-1, neuropilin-1; pDCs, plasmocytoid dendritic cells; PlGF, placenta growth factor, siRNA, small interfering RNA; sNRP-1, soluble NRP-1; Tregs, Tregulatory cells; VEGF, vascular endothelial growth factor; VEGFRs, VEGF receptors.
